# Pathophysiology and Prevention of Manual-Ventilation-Induced Lung Injury (MVILI)

**DOI:** 10.3390/pathophysiology31040042

**Published:** 2024-10-12

**Authors:** Luke A. White, Steven A. Conrad, Jonathan Steven Alexander

**Affiliations:** 1 Department of Molecular and Cellular Physiology, LSU Health Shreveport, Shreveport, LA 71103, USA; luke.white@lsuhs.edu; 2 Department of Internal Medicine, LSU Health Shreveport, Shreveport, LA 71103, USA; steven.conrad@lsuhs.edu; 3 Department of Emergency Medicine, LSU Health Shreveport, Shreveport, LA 71103, USA; 4 Department of Pediatrics, LSU Health Shreveport, Shreveport, LA 71103, USA; 5 Department of Neurology, LSU Health Shreveport, Shreveport, LA 71103, USA

**Keywords:** manual, ventilation, hyperventilation, lung injury, pulmonary injury, resuscitation, CPR, emergency, bag-valve mask, Ambu

## Abstract

Manual ventilation, most commonly with a bag-valve mask, is a form of short-term ventilation used during resuscitative efforts in emergent and out-of-hospital scenarios. However, compared to mechanical ventilation, manual ventilation is an operator-dependent skill that is less well controlled and is highly subject to providing inappropriate ventilation to the patient. This article first reviews recent manual ventilation guidelines set forth by the American Heart Association and European Resuscitation Council for providing appropriate manual ventilation parameters (e.g., tidal volume and respiratory rate) in different patient populations in the setting of cardiopulmonary resuscitation. There is then a brief review of clinical and manikin-based studies that demonstrate healthcare providers routinely hyperventilate patients during manual ventilation, particularly in emergent scenarios. A discussion of the possible mechanisms of injury that can occur during inappropriate manual hyperventilation follows, including adverse hemodynamic alterations and lung injury such as acute barotrauma, gastric regurgitation and aspiration, and the possibility of a subacute, inflammatory-driven lung injury. Together, these injurious processes are described as manual-ventilation-induced lung injury (MVILI). This review concludes with a discussion that highlights recent progress in techniques and technologies for minimizing manual hyperventilation and MVILI, with a particular emphasis on tidal-volume feedback devices.

## 1. Introduction

There are numerous clinical scenarios in which ventilation is accomplished directly by healthcare professionals (manual ventilation) rather than by a machine (mechanical ventilation) [1]. Indications for manual ventilation use include (1) emergency ventilation where mechanical ventilation is not feasible (during either inpatient or out-of-hospital cardiopulmonary resuscitation, or CPR) or (2) short-term ventilatory support (e.g., during a tracheal intubation procedure prior to mechanical ventilator connection or transporting patients around the hospital or to another facility). Although a life-sustaining maneuver, manual ventilation is not as precisely controlled as mechanical ventilation, increasing the risk of inappropriate, even injurious, ventilation [2]. Despite specific guidelines set forth by groups such as the American Heart Association (AHA) and European Resuscitation Council (ERC), unrecognized manual hyperventilation commonly occurs, particularly during resuscitation efforts, and can lead to acute, life-threatening pneumatic and hemodynamic changes. Acute pulmonary injury can also occur from gastric regurgitation and subsequent aspiration. Additionally, a more insidious, sub-barotraumatic lung injury may also occur, similar in pathophysiology to ventilator-induced lung injury (VILI), if manual ventilation occurs for a sufficiently long time. To minimize the risk of manual-ventilation-induced lung injury (MVILI), recent advancements have been made in devices utilized during manual ventilation, most notably tidal-volume feedback devices.

## 2. Manual Ventilation

The most common method for manual ventilation utilizes an inflating/deflating bag connected through a one-way valve either to a mask (bag-valve mask, BVM) or endotracheal tube. Ventilation is achieved when the healthcare provider squeezes the bag, forcing air from the bag and into the patient’s lungs, causing inspiration. With the release of the bag squeeze, air then moves out of the patient’s lungs and into the atmosphere via the nonrebreathing valve. Reinflation of the bag between bag squeezes may or may not require an external pressurized gas source, and those that do not are called self-inflating bags. This process of squeezing and releasing the bag is repeated, and the patient is manually ventilated by the healthcare provider [1].

Although simple in concept, manual ventilation can be difficult to perform correctly [3]. When used with a mask applied to the face, appropriate mask size selection and good hand placement are necessary to create a tight seal between the mask and patient for effective ventilation. Airway patency and evidence of ventilation (such as seeing the patient’s chest rise and fall) should be assessed continuously. Appropriate tidal volumes and respiratory rates for the patient’s age and size should also be delivered to prevent both inadequate ventilation (if bagged too shallowly or slowly) and avoid injury (if bagged too aggressively or quickly). Guidelines for optimal manual ventilation parameters during the resuscitation of adult, pediatric, and neonatal patients have been published from groups such as the American Heart Association (AHA) and the European Resuscitation Council (ERC) and are summarized in Table 1 [4,5,6,7,8,9].

## 3. Manual Hyperventilation

Despite these recommendations, manikin studies have demonstrated that healthcare personnel do not routinely maintain these guidelines regarding tidal volume and respiratory rate delivery during manual ventilation. In one manikin study, 20 ambulance personnel bagged an adult manikin at a mean tidal volume 746 ± 221 mL, 24% higher than the study’s defined target tidal-volume upper bound of 600 mL [10]. Another manikin study from 140 participants including first-aid workers, nurses, and physicians bagged a manikin at an average inspiratory tidal volume of 590 ± 193 mL at a rate of 24 ± 9 breaths/min [11]. In this study, a mask was used to interface between the bag and the manikin’s test lung, likely leading to an inadvertent leak, and the mean delivered tidal volume was 334 ± 124 mL. Excessive ventilation, defined in the study as tidal volumes greater than 600 mL and respiratory rates above 15 breaths/min averaged over a 1 min sliding window throughout the 5 min test, occurred in over 79% of tests For manual ventilation of pediatric patients, one study simulating 72 inpatient pediatric CPR events including pediatric resident and intern physicians, respiratory therapists, and nurses found that BVM hyperventilation occurred during every simulation [12]. The mean respiratory rate was of 41 ± 12 breaths/min, double the study’s target ventilation rate of 20 breaths/min. For manual ventilation of neonatal patients, one manikin study found that the average inspiratory tidal volume was 96 ± 19 mL with a five-fingered grip technique and 88 ± 16 mL with a two-fingered grip at rates of 43 ± 16 and 47 ± 17 breaths/min, respectively [13]. Expiratory tidal volumes were also measured at 62 ± 24 mL and 59 ± 17 mL, respectively. In this study, there were no criteria for excessive ventilation since the study’s endpoint was comparing five- versus two-fingered grip techniques. Nonetheless, even assuming a simulated large 5 kg neonate (a birthweight greater than 99.9% of the recorded birthweights for the United States in 2019 [14]), the measured inspiratory and expiratory tidal volumes were nearly 50% above the ERC’s upper limit for its recommended tidal volume of 8 mL/kg (a tidal volume of 40 mL) [9]. Furthermore, these volumes are nearly three times greater than the recommended tidal volume for low-birthweight neonates (defined as a birthweight less than 2.5 kg, giving a recommended tidal volume of 20 mL) [14], which is one of the most important risk factors associated with neonatal respiratory distress syndrome and the need for emergent manual ventilation [15].

Clinical studies involving real-life resuscitation scenarios have also documented the alarming tendency for healthcare personnel to hyperventilate. In 2004, a study was published where the quality of out-of-hospital CPR performed by emergency medical personnel was observed [16,17]. After seven cases, they noted that the average delivered respiratory rate was 37 ± 4 breaths/min, over three times the target respiratory rate of 12 breaths/min. After a retraining of the personnel, the respiratory rate dropped to 22 ± 3 breaths/min in the subsequent six cases, which was still nearly double the target respiratory rate. The authors concluded that hyperventilation during emergency manual ventilation in a pre-hospital setting is a common problem. In a 2006 study, respiratory variables were measured during resuscitation attempts with 12 patients in an in-hospital setting of an emergency department [18]. The median tidal volume delivered was 619 mL (range of 374 to 923 mL) at a median rate of 21 breaths/min (range of 7 to 37 breaths/min).

## 4. Manual-Ventilation-Induced Lung Injury (MVILI)

**Figure 1:**
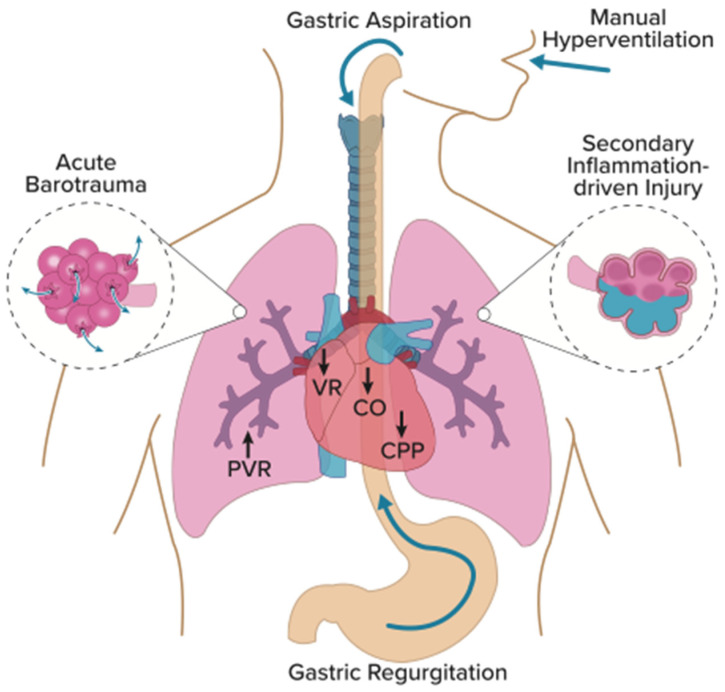
Mechanisms of MVILI. Manual hyperventilation increases intrathoracic pressure leading to adverse hemodynamic changes, including decreased venous return (VR), cardiac output (CO), and coronary perfusion pressure (CPP). Additionally, increased inspiratory pressures can open the lower esophageal sphincter, causing gastric insufflation, regurgitation, and aspiration. Vigorous manual ventilation may also lead to acute barotrauma (pneumothorax, pneumomediastinum, pneumoperitoneum, and subcutaneous emphysema) and possibly a sub-barotraumatic, inflammation-driven injury lung injury. Graphic design by David Wright.

The above studies demonstrate that healthcare personnel routinely hyperventilate patients during manual ventilation, events that can have deleterious effects on the resuscitated patient. Previous research has focused on two mechanisms of manual hyperventilation injury: (1) adverse hemodynamic changes and (2) gastric insufflation (in non-intubated, unprotected airways) leading to aspiration lung injury. Evidence from several case studies (particularly in intubated, closed-circuit airways) also suggests gross barotrauma, such as pneumothorax, pneumomediastinum, and pneumoperitoneum, as another important mechanism of lung injury. Finally, a more insidious secondary, inflammation-driven lung injury has been surmised to occur, similar in mechanism to what has been extensively studied as ventilator-induced lung injury (VILI), although experimental and clinical evidence for this pathophysiology is scarce. Collectively, these mechanisms of lung injury can be classified as manual-ventilation-induced lung injury or MVILI (Figure 1).

### 4.1. Adverse Hemodynamic Changes

Because the thoracic cavity is occupied by both the lungs and the heart, alterations to intrathoracic pressure through positive pressure mechanical ventilation can affect cardiac dynamics [19,20,21]. Aufderheide et al. experimentally demonstrated in a porcine model how hyperventilation (from 12 to 30 breaths/min) can increase mean intrathoracic pressure and decrease coronary perfusion pressure (experimentally measured as the difference between aortic diastolic pressure and right atrial diastolic pressure) during CPR [16,17]. Furthermore, return of spontaneous circulation occurred after biphasic shock in nearly all (six of seven pigs, 86%) pigs ventilated at 12 breaths/min, while only one of seven (14%) of pigs ventilated at 30 breaths/min was successfully resuscitated. The authors speculated that the increase in intrathoracic pressure led to a decrease in venous return, reducing right ventricular preload and cardiac output. They also speculated that this increased intrathoracic pressure would reduce left ventricular cardiac output. Additionally, the decrease in coronary perfusion pressure would lead to decreased myocardial blood flow, a vital component for a successful return of spontaneous circulation. The authors expressed concern that these hemodynamic changes from manual hyperventilation during resuscitation attempts could severely impact outcomes and survival.

### 4.2. Gastric Regurgitation and Aspiration

Some forms of manual ventilation, in particular the use of a mask, exposes the patient’s esophagus to increased airway pressure. At high-enough pressures, the lower esophageal sphincter (LES) may open, leading to gastric insufflation. Subsequent regurgitation of gastric contents back up through the esophagus and into the airway may then occur, leading to a pulmonary aspiration injury.

In 1961, Ruben and colleagues reported the static airway pressures necessary to induce gastric insufflation in 20 patients [22]. They found that pressures below 15 cm H_2_O rarely induced gastric inflation while pressures greater than 25 cm H_2_O induced gastric inflation in most patients. In a porcine model of cardiac arrest, it was shown that the airway pressure necessary to open the LES decreased from 20.6 ± 2.8 cm H_2_O at baseline to 5.6 ± 4.6 cm H_2_O after 5 min of cardiac arrest [23]. The mechanisms underlying these reductions in LES sphincter opening pressure are not clear. A later case series on three patients undergoing withdrawal of life support demonstrated a similar decrease in LES opening pressure from approximately 20 cm H_2_O to 5 cm H_2_O during the first few minutes of cardiac arrest [24]. These data suggest that decreased lower esophageal sphincter tone (which may exist during cardiac arrest and other stresses) could increase susceptibility to gastric regurgitation during BVM ventilation of cardiac arrest patients. Indeed, one study found that gastric regurgitation occurred during manual ventilation in 12.4% of the 466 CPR patients ventilated with a BVM [25].

The airway pressures generated during an inspiration for a given patient during BVM ventilation is inextricably tied to the airflow rate and thoracic compliance. An increase in tidal volume or decrease in inspiratory time could lead to greater generated airflow rates and increased airway pressures. Thus, BVM hyperventilation—where tidal volumes increases and inspiratory times decreases—increases one’s susceptibility to gastric regurgitation and lung aspiration [26,27].

### 4.3. Barotrauma

Mechanical ventilation with inappropriate ventilatory parameters can lead to a primary mechanical injury in the bronchotracheal tree. If severe enough, this can lead to a hyperacute pressure injury, including pneumothorax, pneumomediastinum, and pneumoperitoneum. Such injury has been historically classified as barotrauma. Several case studies have documented the incidence of barotrauma due to injurious manual hyperventilation. Although some reported barotraumatic injuries were the result of faulty equipment, such as a misassembled BVM [28,29], there are other cases where manual hyperventilation performed directly by the healthcare provider likely induced the injury [30,31,32]. However, beyond case reports, there is a lack of larger clinical studies on acute barotrauma in manually ventilated patients.

### 4.4. Sub-Barotraumatic and Secondary Inflammation-Driven Injury

In VILI, a link to primary mechanical damage that triggers a secondary inflammation-driven injury is well established [33]. The mechanical damage is induced by lung overdistension (volutrauma) and cyclic opening and closing of collapsible lung units (atelectrauma). These types of injurious mechanical ventilation that do not induce an acute barotraumatic episode can still lead to damage through this secondary inflammation-driven injury. However, whether BVM hyperventilation can also induce a sub-barotraumatic, secondary inflammatory injury is not known. One may reasonably speculate that the same pathophysiologic mechanisms that drive VILI could also apply in MVILI.

A key difference between manual and mechanical ventilation is the timeframe during which a patient is subjected to each method of ventilation. It is not uncommon for patients to be ventilated with a mechanical ventilator for days to weeks, potentially subjecting them to sub-barotraumatic levels of injurious ventilation for an extended duration. Manual ventilation, on the other hand, may only be performed for minutes or, in some extreme cases of emergency surge crises, hours at a time [34,35]. Nevertheless, manual ventilation is far less well controlled, lacking the precision and safety alarms mechanical ventilation offers. A crucial question that remains unanswered is whether manual hyperventilation realistically occurs over a long-enough timeframe to induce this secondary inflammatory injury, and experimental and clinical data are lacking.

Blackburn et al. performed manual hyperventilation in pigs for one hour following moderate hemorrhaging to simulate resuscitation in a trauma patient [36]. After 24 h post-hemorrhage, signs of secondary inflammatory injury were assessed. Plasma pro-inflammatory cytokines IL-1β and IL-2 were only significantly elevated in the maximal hyperventilation group (750 mL tidal volume at 20 breaths/min) at 4 h post-hemorrhage, which returned to baseline. Lung wet-to-dry-weight ratios were similar in all three groups, and no significant differences in pulmonary congestion or presence of thrombi were noted on histological examination. These data suggest that a substantial secondary inflammatory injury to the lungs may not occur in moderate hemorrhage and manual hyperventilation. Other scenarios and models could yield differing results, and the group noted that models that incorporate underlying pulmonary disease may predispose the lungs to further injury by manual hyperventilation. Of particular interest would be hyperventilation in a model of neonatal respiratory distress, as the mismatch between recommended and delivered tidal volumes has the potential to be much higher compared to adult models of manual hyperventilation.

### 4.5. Current Knowledge Gaps

Although VILI is a well-studied and accepted pathology that can occur during mechanical ventilation, MVILI, in contrast, is vastly understudied. The lack of large, high-quality clinical studies on MVILI makes even its incidence, particularly during resuscitation, currently unknown. For this reason, it is unclear whether MVILI is either greatly underdiagnosed or a mostly theoretical concept without much clinical correlation. Nevertheless, as described above, expert opinion and society guidelines insist on the minimization of manual hyperventilation to decrease the possibility of MVILI’s pathophysiological processes.

Other knowledge gaps on manual hyperventilation and MVILI exist in the literature. For instance, scenarios where persistent manual hyperventilation outweighs the risks of MVILI are not well studied, particularly in conjunction with resuscitation attempts. Similar to therapeutic mechanical hyperventilation, there may be specific scenarios where manual hyperventilation may be preferred in select patients, such as in certain cases of traumatic brain injury [37,38] or during a newborn pulmonary hypertensive crisis [6,39]. Additionally, patient–mechanical ventilation dyssynchrony, where phases of a patient’s respiratory attempts do not match with the supported mechanical ventilation, is a well-documented adverse interaction that can occur between patients and mechanical ventilators [40,41]. One may suspect patient–manual ventilation dyssynchrony to also exist, and expert opinion has warned against it [42,43], but no studies that investigated its prevalence in scenarios of resuscitation have been found.

Another gap of knowledge in the literature is the appropriate application of positive end expiratory pressure, or PEEP, during manual ventilation. Mechanical ventilation can lead to improved gas exchange while simultaneously reducing the risk of atelectrauma, i.e., injury to the lungs from cyclical opening and closing of alveoli [33]. Similarly, maintenance of PEEP during manual ventilation with a PEEP valve, an adjustable one-way valve fitted to the exhalation arm of the bag, can be utilized to prevent alveolar collapse between bag squeezes [44]. However, PEEP pressures that are too high can increase the risk of certain MVILI pathophysiologies, particularly gastric insufflation and aspiration in non-intubated patients and worsening hemodynamics due to a continued increase in intrathoracic pressure, as described above. Optimal PEEP during resuscitation, during either mechanical or manual ventilation, has not been well defined, and guidelines for PEEP during resuscitation are either absent or vague for adult and pediatric populations [4,5,6,7]. The most specific recommendation comes from the AHA and ERC neonatal guidelines, recommending that PEEP be maintained around 5 to 6 cm H_2_O, especially in preterm neonates [8,9].

The application of mouth-to-mouth or mouth-to-nose rescue breathing is another form of manual ventilation that can be provided by a rescuer, particularly if no other form of ventilation is available. It may be suspected that rescue breathing may not only be effective at providing adequate oxygenation and ventilation [45], but that it may provide a means of manual ventilation that is less injurious than using a bag. As the eighteenth-century physician John Fothergill stated, “the lungs of one man may bear, without injury, as great a force as those of another man can exert” [46]. Interestingly, no studies that investigated the extent to which hyperventilation occurs with rescue breathing during resuscitation could be found. Given that rescue breathing is not a discouraged form of manual ventilation in adult and pediatric patients [4,5,6,7], this remains a large knowledge gap in the current literature.

## 5. Therapies for MVILI Reduction

Methods for minimizing manual hyperinflation to limit MVILI while simultaneously ensuring adequate ventilation would improve the safety and efficacy of manual ventilation. Specifically, the delivery of proper tidal volumes at an appropriate ventilatory rate to the patient is of primary importance. Attempts have been made to improve bagging through several methods, including (1) changes in bag size and design optimization, (2) pressure manometry monitoring, and (3) direct tidal-volume feedback.

### 5.1. Bag Size and Design Optimization

One of the simplest methods for reducing manual hyperventilation is to use smaller bags. Common adult self-inflating bags range in their total volume from 1450 to 1650 mL [47,48,49,50]. Doerges et al. found that delivered tidal volumes can be reduced from 334 ± 125 mL with an adult bag and mask to 256 ± 77 mL with a pediatric bag [51]. More recently, Dafilou and colleagues found that tidal volumes could be reduced from 808 ± 160 mL with an adult bag down to 631 ± 85 mL with a pediatric bag when used with an endotracheal tube [52]. Wenzel et al. found that the use of pediatric bags during anesthesia induction prior to surgery reduced exhaled tidal volumes from 779 ± 122 mL to 365 ± 55 mL [53]. The discrepancy of delivered tidal volumes between the first and subsequent two studies is likely due to the loss of delivered tidal volume due to mask leak.

Utilizing different bagging techniques, such as bagging with one versus two hands, can also influence tidal-volume delivery [54]. To reduce this variation, Cho et al. placed specific markings on the bag to indicate where fingers should be placed to facilitate non-injurious and consistent tidal-volume delivery [55]. Among the 83 healthcare personnel who participated in the manikin study, the percentage of individuals bagging at tidal volumes between the target volumes of 500 and 600 mL increased from 17% to 93% when the marked bags were used. Additionally, the standard deviation of the delivered tidal volumes with the marked bag group was lower (24 mL) compared to the unmarked bag group (95 mL), consistent with a reduction in inter-user variability. Based on these findings, the choice of correct bag size is highly sensitive to (1) the presence and extent of an unintended leak, likely due to a poor mask seal, and (2) the methodology by which the bag is squeezed.

Another method for providing more appropriate manual ventilation is to redesign the self-inflating bag altogether. Merrell et al. recently tested a novel BVM called the Butterfly BVM, which allows for the adjustment of valves on the device to provide a specific peak inspiratory pressure, tidal volume, and bag reinflation rate to provide more consistent ventilation [56]. Utilizing adult and pediatric manikins, the mean tidal volume provided by participants were more consistently within the target range (defined as 4 to 8 mL/kg; 280 to 560 mL for a 70 kg adult and 48 to 112 mL for a 12–14 kg pediatric patient) with the Butterfly BVM (351 ± 50 mL for adult patients; 90 ± 18 mL for pediatric patients) compared to using a standard BVM (629 ± 94 mL for adult patients; 181 ± 57 mL for pediatric patients).

### 5.2. Pressure Manometry Monitoring

For a given patient, the applied airway pressure and subsequent administered tidal volume are related through thoracic compliance. All other parameters being identical, an increase in peak airway pressure during the delivery of a breath would result in an increase in delivered tidal volume [57]. Pressure manometers are simple and inexpensive devices that measure air pressure. Because of the relationship between volume and pressure, pressure manometers can potentially be used as a surrogate index for tidal-volume delivery, where limiting the pressure below a certain threshold could reduce manual hyperventilation.

Manikin studies that examine the utility of pressure manometers during manual ventilation have provided equivocal results. An early neonatal manikin study by Zmora and Merritt showed an increase in success rates of achieving two target peak inspiratory pressures (with the ranges of 15 ± 2 and 30 ± 2 cm H_2_O) with lower variations when manually ventilating with a manometer [58]. Karsdon et al. performed a similar study and also demonstrated a decrease in variation with peak inspiratory pressure at a target peak inspiratory pressure of 15 cm H_2_O; however, there was no difference with manometer use at the higher target peak inspiratory pressure of 25 cm H_2_O [59]. O’Donnell et al. more recently found that manometer use on a neonatal manikin did not improve delivered peak inspiratory pressure accuracy (target pressure of 25 cm H_2_O) [60]. Furthermore, they found that manometer use did not alter delivered tidal volume or percent leak from the facemask during bagging.

Beyond whether pressure manometry can improve peak inspiratory pressure accuracy, a more fundamental problem emerges. The thoracic compliance relating peak airway pressure and tidal-volume delivery is both patient- and condition-specific, depending upon several factors including the patient’s lung volumes, pulmonary compliance, airway resistance, and any underlying pulmonary or chest wall disease [61]. One cannot measure pressure and simply deduce delivered tidal volume without prior knowledge of these other parameters. This is especially important during neonatal transition, where pulmonary mechanics change rapidly [62]. Kattwinkel et al. showed in a manikin model of dynamic lung compliance that tidal-volume monitoring outperformed pressure monitoring in achieving the target tidal volume (4 to 6 mL/kg) [63]. The authors in this study suggested that devices providing tidal-volume feedback should routinely be included in neonatal resuscitation equipment.

### 5.3. Tidal-Volume Feedback

**Figure 2:**
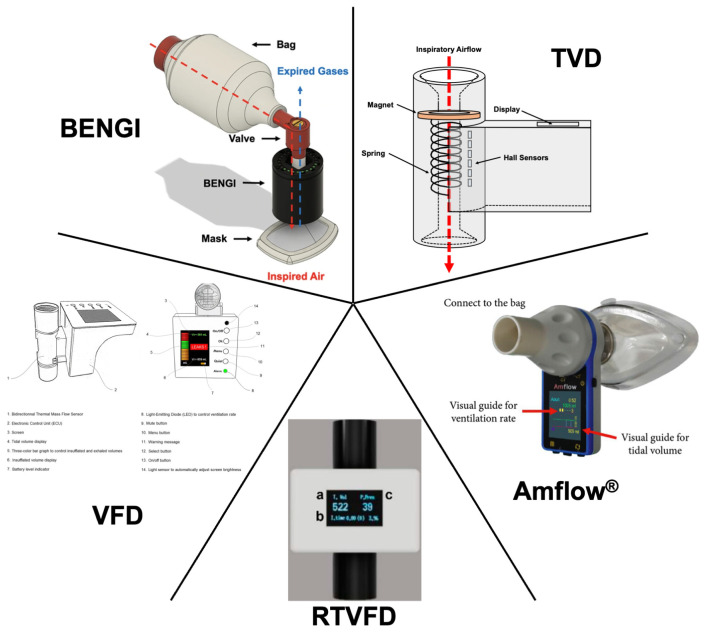
Tidal-volume feedback devices. Authors’ rendering of the TVD based on [67]. Cropped and edited images of the Amflow^®^ [68], RTVFD (a, tidal volume; b, inspiration time; c, peak pressure) [69], VFD [70], and BENGI [71] reproduced under Creative Commons Attribution Non-Commercial License 4.0.

Recently, low-cost and portable devices that provide instantaneous feedback on delivered tidal volumes during manual ventilation have been developed, termed tidal-volume feedback devices (Figure 2). These devices are inserted between the bag and mask, endotracheal tube, or other advanced airway device, measuring the airflow from the bag to the patient; from this measurement, the tidal volume can be calculated and immediately reported to the healthcare provider utilizing an onboard display. Other useful parameters, such as respiratory rate or measured percent air leaks, may also be displayed. Some of these devices also incorporate respiratory rate guidance with a countdown timer, as it has been shown that metronome guidance improves manual ventilation respiratory rates [64,65,66].

In 2017, You et al. reported the development and testing of the first of such devices in the literature, the tidal-volume feedback (TVD) device [67]. The TVD prototype contains a magnet connected to a compression spring placed within the air conduit of the device. When air is pushed through the device by bag compression, the magnet and spring are displaced by a distance proportional to the airflow, and six highly sensitive Hall sensors detect this displacement. A microcontroller utilizing specified numerical equations determines the delivered tidal volume, which is directly displayed to the user during each delivered breath. The device, when connected between a mechanical ventilator and test lung, provided accurate measurements of tidal volume (mean difference 1.02 ± 0.15 mL). In a manikin study, pooled optimal tidal-volume delivery (defined as 420 to 490 mL) frequency increased from 32% without the TVD to 84% with the TVD. Although shown to be more accurate and efficacious, the TVD does not allow for airflow back through the device, necessitating a second one-way expiratory valve.

The Amflow^®^, developed by MEDICION and tested by Kim and colleagues, is a handheld device that utilizes a turbine and infrared sensor to measure airflow and calculate delivered tidal volume [68]. The device uses an onboard display to output measured tidal volume graphically, as a bar whose size is dependent on the proportion of a set target tidal volume. A countdown timer is also displayed to guide the ventilation rate at a defined rate. A manikin study demonstrated improvements to pooled tidal-volume delivery in scenarios of cardiac arrest (target tidal volume of 450–550 mL achieved; 85% with Amflow^®^ versus 41% without Amflow^®^) and acute respiratory distress syndrome (315–385 mL; 59% with versus 24% without) with no difference in a head trauma scenario (630–770 mL; 66% with versus 68% without). Pooled respiratory rates also improved in all three scenarios (within 0.1 breaths/min of target rates of 10, 20, and 15 breaths/min for cardiac arrest, acute respiratory distress syndrome, and head trauma scenarios, respectively).

The Real-Time Ventilation Feedback Device (RTVFD), developed and tested by Heo et al., utilizes a mass flow sensor to measure flow rate and calculate delivered tidal volume, which is numerically displayed on an onboard screen [69]. After an inspiration is detected, a countdown timer is also displayed to indicate when the next breath should be delivered to achieve ventilation at a set respiratory rate. The device can also be connected to smart devices via Bluetooth to display bagging pressures and air volume delivery in real time. The efficacy of the RTVFD was tested with both an adult and pediatric bag in a two-scenario manikin study. In the adult scenario, RTVFD use improved delivery of optimal tidal volume (defined as 420 to 480 mL) and respiratory rate (defined as one breath delivered every 6 to 8 s) from 18% to 47% and from 50% to 96%, respectively. Similarly, RTVFD use improved optimal tidal volume (120 to 180 mL) and respiratory rate (again, one breath delivered every 6 to 8 s) delivery in the pediatric scenario from 73% to 90% and 57% to 96%, respectively.

Khoury et al. developed the Ventilation Feedback Device (VFD) [70]. The VFD is a handheld device that utilizes a commercially available and disposable flow sensor that connects to an electronic unit. The VFD provides visual (via an onboard display) and audible (through an onboard speaker) tidal-volume and respiratory rate feedback, along with information on inspiratory/expiratory time and percent air leakage. The user can set a target range of ideal tidal volumes based on the patient’s estimated ideal body weight (6 to 7 mL/kg), and the VFD targets a specific ventilation rate for the patient, determined based on a calculated and measured expiratory time constant. Furthermore, the VFD provides visual warning messages to the user if inadequate ventilation or a high level of air leak is detected. A manikin study with 40 participants demonstrated an improvement in acceptable ventilation (defined as 300 to 600 mL at 8 to 15 breaths/min) by both basic-life-support-certified (15% without versus 90% with VFD) and advanced-life-support-certified (15% without versus 85% with VFD) healthcare personnel.

More recently, Maxey et al. have developed the BVM Emergency Narration Guided Instrument (BENGI) [71]. The BENGI utilizes simplified audiovisual cues in the form of LEDs and pre-recorded voice-guided instructions to guide the user on appropriate bagging. The LEDs are arranged in a ring and sequentially light up as one approaches the target tidal volume, which can be set as appropriate for adult, pediatric, and neonatal patients. Audio cues instruct the user when to initiate a bag squeeze and alerts the user if a bag squeeze is performed too slowly or quickly. Another audio cue alerts the user if a substantial leak from the mask is detected. A randomized crossover manikin study demonstrated that BENGI use resulted in manual ventilation that more closely and consistently approached target tidal volumes and respiratory rates in adult (500 and 750 mL at 10 breaths/min), pediatric (300 mL at 10 breaths/min), and neonatal (20 mL at 60 breaths/min) scenarios [72] (in press).

## 6. Summary

Manual ventilation remains a very common emergency ventilation procedure that has undergone very few changes despite its widespread implementation nearly 70 years ago. Delivery of manual ventilation is highly uncontrolled, and the efficacy and safety of its performance is based on the user’s individual training, experience, and intuition without objective feedback indicating appropriate ventilation. Unfortunately, several manikin studies and clinical observations have shown that manual ventilation users frequently hyperventilate while bagging. Manual hyperventilation can lead to adverse, even life-threatening, effects. These include altered hemodynamics, increased risk of gastric regurgitation and aspiration, barotrauma, and possibly a sub-barotraumatic inflammation-driven injury. Recently, advancements have been made to make manual ventilation less subjective. Most promising are handheld real-time tidal-volume monitoring devices. However, these devices have not been widely implemented, likely due to the lack of high-quality clinical data that demonstrate their efficacy. Nevertheless, the adoption of such devices has the potential to improve manual ventilation safety and efficacy.

## Figures and Tables

**Table 1:** Summary of AHA and ERC guidelines for ventilation during resuscitation. “+” denotes “with” and “−” indicates “without” advanced airway (AA) or initiation of chest compressions (CC)**.** Where applicable, “:” is used to denote “chest-compression-to-delivered-breath ratio” during resuscitation (for example, 30:2 would imply 30 chest compressions followed by the administration of 2 breaths). PIP, peak inspiratory pressure; RR, respiratory rate; V_T_, tidal volume; yo, year old.

			AHA Recommendations	ERC Recommendations
**Adult**	**V_T_**		500–600 mL or chest rise	500–600 mL or chest rise
	**RR**	**−AA**	30:2 or 10 breaths/min	30:2 or 10 breaths/min
		**+AA**	10 breaths/min	10 breaths/min
**Pediatric**	**V_T_**		No specific recommendation	6–8 mL/kg, or chest rise
	**RR**	**−AA**	30:2 (1 rescuer) or 15:2 (≥2 rescuers)	15:2
		**+AA**	20–30 breaths/min	25 (infants), 20 (1–8 yo), 15 (8–12) yo, or 10 (>12 yo) breaths/min
**Neonatal**	**PIP**		Up to 30 (term) or 20–25 cm H_2_O (preterm) but occasionally higher if needed	30 (term) or 25 cm H_2_O (preterm), or 5–8 mL/kg if being monitored
	**RR**	**−CC**	40–60 breaths/min	30 breaths/min
		**+CC**	3:1	3:1
